# Varicella zoster virus meningitis with a dermatomal rash in immunocompetent hosts: a systematic review and novel case report

**DOI:** 10.1007/s13365-026-01345-3

**Published:** 2026-10-08

**Authors:** Nathaniel B. Dusseau, Aizaz Ali, Ahmed Ba Theeb, Joyeta Razzaque, Sumayya Naz

**Affiliations:** 1 University of Toledo College of Medicine and Life Sciences, 3000 Arlington Rd, Toledo, OH 43614 USA; 2 Department of Neurology, University of Toledo, Toledo, OH USA

**Keywords:** Varicella zoster virus, Meningitis, Herpesvirus, Rash

## Abstract

Varicella zoster virus (VZV) reactivation with central nervous system (CNS) involvement is an uncommon but important cause of lymphocytic meningitis in both immunocompromised and immunocompetent individuals. While cranial presentations, especially V1 involvement, are well documented, spinal-dermatomal VZV meningitis is rarely reported. We conducted a systematic review of PubMed, Scopus, and Embase using PRISMA guidelines to identify all cases of laboratory-confirmed VZV meningitis or encephalitis with a dermatomal rash in immunocompetent adults affecting spinal levels C1 and below. Data extracted included demographics, dermatome distribution, CSF findings, treatment, and outcomes. We also report a novel case from our institution involving isolated C4–C5 involvement. Seventeen total cases met inclusion, including our 44-year-old male patient who presented with fever, severe headache, and a right-sided vesicular rash in the C4–C5 dermatomes. He was initially misdiagnosed with a tick-borne illness. Mean CSF values across all cases were WBC count 382/µL (SD ± 270), protein 177 mg/dL (SD ± 106), and glucose 52.7 mg/dL (SD ± 15.8). IV acyclovir was used in 15 of 17 cases, with oral acyclovir (41%) or valacyclovir (29%) as the most common step-down therapies. Mean treatment duration was 14 days, and average length of hospital stay was 6.4 days. All patients survived and made a full recovery. Spinal-dermatomal VZV meningitis can occur in immunocompetent adults, including cases with cervical involvement. Early recognition of the rash pattern and timely PCR testing are essential for accurate diagnosis and prompt antiviral therapy, which is associated with favorable outcomes.

## Introduction

Varicella zoster virus (VZV) is a neurotropic alpha-herpesvirus that establishes latency in dorsal root and cranial nerve ganglia following primary infection(Gershon et al. 2015; Gilden et al. 2015; Yawn and Gilden 2013). Reactivation, which classically presents as dermatomal herpes zoster can extend beyond the skin to involve the CNS, causing meningitis, encephalitis, myelitis, or vasculopathy(Kennedy 2023; Nagel et al. 2020). Although ophthalmic involvement and cranial presentations are well described, the intersection of spinal dermatomal rash with meningitis remains less systematically characterized(Gilden et al. 2013; Kennedy and Gershon 2018). Clinicians may overlook spinal dermatomal zoster as an etiologic clue in adults presenting with acute febrile headache, particularly in immunocompetent individuals. In this manuscript, we performed a systematic review of the literature to find all reported cases of VZV meningitis with a spinal dermatomal vesicular rash presenting in immunocompetent adults. Additionally, we present a novel case of C4–C5 dermatomal VZV meningitis, adding to the anatomic spectrum of the disease.

## Methods

### Systematic review

Using Preferred Reporting Items for Systematic Reviews and Meta-Analyses guidelines (PRISMA) guidelines, a systematic review was conducted to find all manuscripts presenting at least one case of VZV meningitis with a spinal dermatomal vesicular rash in an immunocompetent adult(Moher et al. 2009). PubMed, Scopus, and Embase were searched on July 27th, 2025. Medical Subject Headings used were (((Varicella Zoster Virus) OR (Varicella) OR (VZV) AND (Meningitis) AND (Rash). No restrictions on the range of publication dates were applied. Inclusion criteria included patients with VZV meningitis or encephalitis accompanied by rash in dermatomes C1 and below; V1–V3 and purely cutaneous zoster without CNS involvement were excluded. Patients had to be ≥18 years old and determined to be immunocompetent. Immunocompetence was defined as the absence of HIV infection, hematologic or solid-organ malignancy, transplantation, and immunosuppressive or immunomodulatory therapy, including chronic corticosteroids; patients with HIV infection were considered immunocompromised regardless of viral suppression. For the systematic review, immune status was accepted as reported by the original authors. Confirmation of diagnosis with polymerase chain reaction (PCR) testing was required. Screening and abstraction were performed independently by a single researcher. Titles and abstracts were screened for relevance to VZV central nervous system infection with an associated rash, and full texts were then assessed against the inclusion and exclusion criteria above. Patient-level variables included demographics, immune status, dermatome level and laterality, CSF parameters, diagnostic method, antiviral therapy, and clinical outcome and ranges were computed for CSF parameters, and antiviral usage and outcomes were summarized. Descriptive statistics and creation of the data table was performed using Microsoft Excel (version 16.96.1; Redmond, WA, USA). Calculations included averages, percentages, and standard deviations. A figure was created using the PRISMA flow chart template. Missing data was noted and reported in our results. PRISMA counts were fixed to the search log above.

### Case report

The electronic medical record of the University of Toledo Medical Center was reviewed. Data including demographics, history and physical, past medical history, laboratory results, progress notes, imaging results, and discharge summary were abstracted. A figure was created by photograph. The patient provided written consent for use of their case and accompanying images for publication.

## Results

### Systematic review

**Fig. 1:**
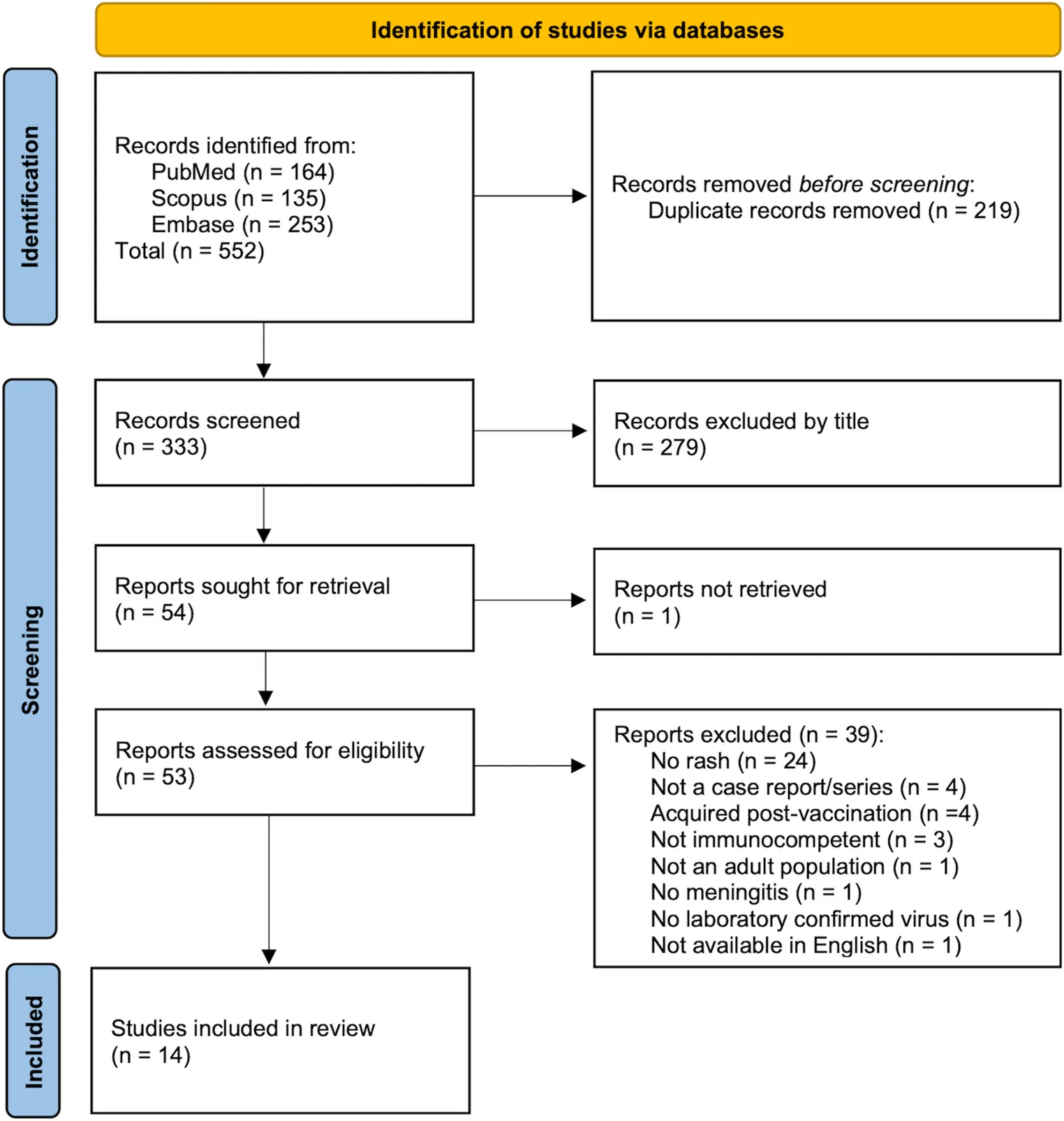
PRISMA flow diagram. A visualization of the systematic review literature search using the PRISMA guidelines (Moher et al. 2009)

Initial search of the literature using our defined MeSH terms identified 552 manuscripts which included 164 from PubMed, 135 from Scopus, and 253 from Embase. After removing 219 duplicates, 333 records remained. Titles/abstracts were screened, and 54 articles were found eligible to have full texts reviewed. After title and abstract screening, 279 records were excluded; of the 54 reports sought for retrieval, one could not be obtained, and the remaining 53 underwent full-text assessment. Thirty-nine reports were excluded (no rash, *n* = 24; not a case report or series, *n* = 4; VZV acquired post-vaccination, *n* = 4; not immunocompetent, *n* = 3; not an adult population, *n* = 1; no meningitis, *n* = 1; no laboratory-confirmed virus, *n* = 1; not available in English, *n* = 1). 14 manuscripts met inclusion criteria. 16 eligible cases were found within those papers(Alataby et al. 2020; Alexakis et al. 2024; Bhandari et al. 2021; Chen et al. 2020; Chirumamilla et al. 2023; Cooper et al. 2013; Faluk et al. 2020; Goyal et al. 2013; Imani et al. 2020; Kangath et al. 2013; Khalil and Hirashima 2021; Khaliq et al. 2018; Kushawaha et al. 2009; Sohal et al. 2020). A PRISMA flowchart can be seen in Fig. 1. Our case report was then added into the pooled analysis, which made a total of 17 patients in this review.

In addition to our cervical dermatomal patients, other rash presentations included one cervicothoracic, six thoracic, one lumbar, and one sacral. The specific dermatome level was not specified in seven cases; however, all included cases described a rash in a spinal (non-cranial) distribution consistent with the inclusion criteria. Mean CSF values across the dataset were: WBC 382/µL (SD ± 270), protein 177 mg/dL (SD ± 106), and glucose 52.7 mg/dL (SD ± 15.8). Intravenous (IV) acyclovir was administered in 15/17 (88%) of all cases. No IV antiviral drug was used in two patients. Oral step-down to acyclovir was most common (seven patients, 41%), followed by valacyclovir (five, 29%). Of note, one patient received oral famciclovir. Four patients (24%) were not discharged with any oral antiviral medication. Mean duration of IV antiviral treatment was 8.3 days (range: 0–21). Oral antiviral treatment lasted an average of 7.0 days (range: 0–14). Overall time of total treatment for VZV meningitis was on average 14 days (range: 3–21). The average length of stay was 6.4 days (range: 3–10). Outcomes were uniformly favorable, with 100% of patients reported as making a full neurological recovery. No deaths were recorded.

## Case report

**Fig. 2:**
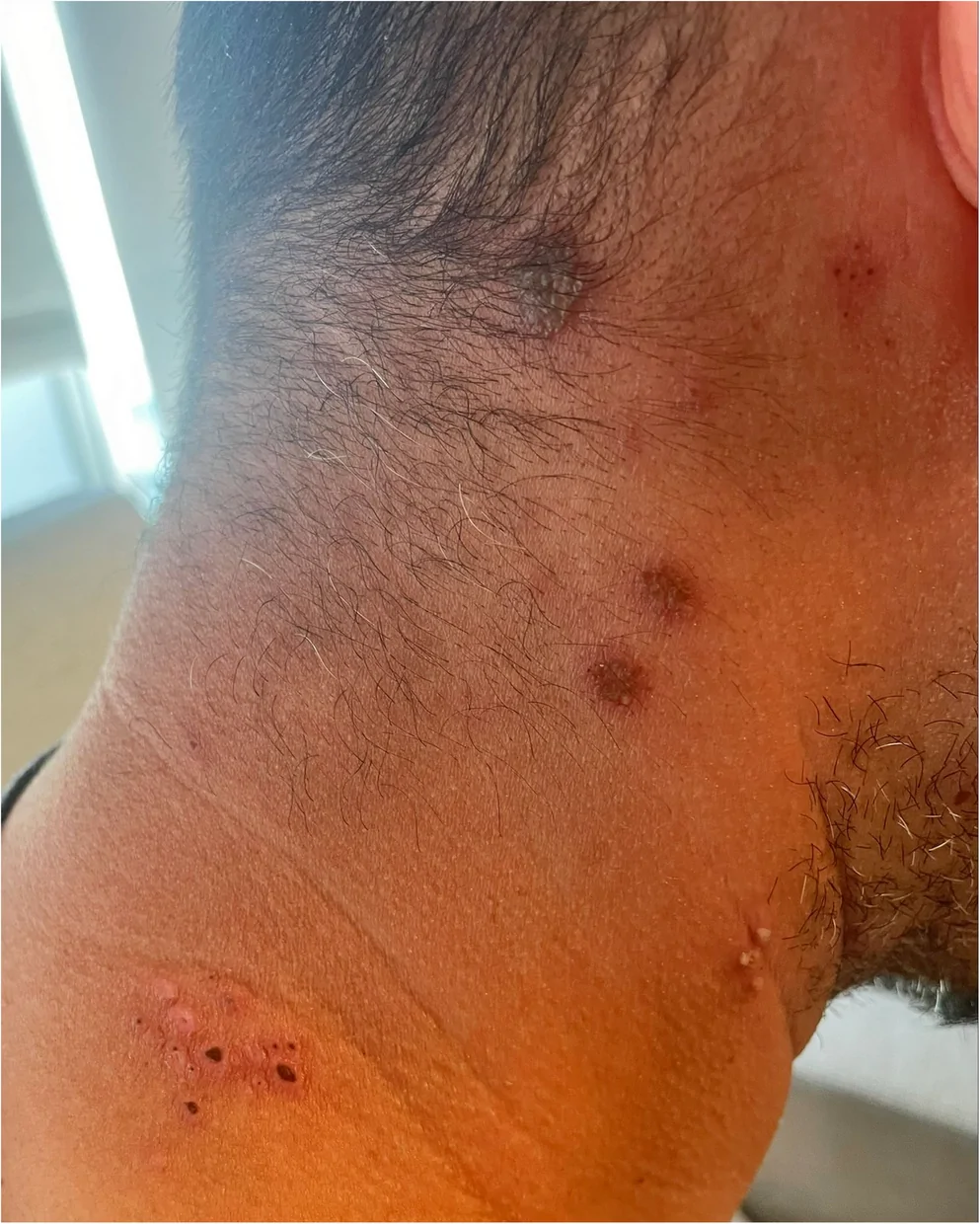
Case Report Rash. Photos of our case report’s right sided C4-C5 vesicular rash from a lateral viewpoint at time of discharge

A 44-year-old immunocompetent man presented to the hospital with six days of flu-like illness including fever, chills, myalgias, as well as a severe, constant retro-orbital/bifrontal headache. He developed a painful vesicular eruption over the right lateral neck and retro-auricular region consistent with C4–C5 dermatomal distribution. Photos of the rash can be seen in Fig. 2. He initially presented to an outside urgent care two days prior to admission and was misdiagnosed with presumed Lyme disease due to occupational tick exposure. At that time the patient was started on doxycycline, and his symptoms progressively worsened. Upon presentation to the emergency department, lumbar puncture revealed CSF WBC count of 298/µL, protein of 179 mg/dL, and glucose of 67 mg/dL. Multiplex PCR was positive for VZV. CT head and MRI brain found no abnormalities. The patient had no history of HIV infection, malignancy, organ transplantation, or immunosuppressive or immunomodulatory therapy and was therefore considered immunocompetent. He had not received the recombinant zoster vaccine and, at 44 years of age, was below the currently recommended age for vaccination.

The patient received intravenous acyclovir 10 mg/kg every 8 h for four days under airborne/contact precautions. Rash lesions crusted and headache resolved, and the patient was subsequently discharged neurologically intact with full recovery on day four of his hospital stay. Upon discharge, he was transitioned to a 10-day course of valacyclovir 1 g three times daily, completing a 14-day total antiviral course. At a follow-up visit two weeks after discharge, the patient remained symptom free, and all lesions were fully healed. This case represents the first purely cervical (C4–C5) dermatomal VZV meningitis in our dataset and illustrates a common diagnostic pitfall: initial misclassification as tick-borne disease. The full dataset of the 17 patients can be found in Table 1.

**Table 1:** Patient dataset. Results of a systematic review for cases of immunocompetent adults with laboratory-confirmed VZV meningitis and dermatomal vesicular rash including demographics, dermatomal distribution, CSF analysis results, treatment details, and patient outcomes

Source	Age	Sex	Past medical history	Dermatome level	CSF opening pressure (mmHg)	CSF WBC count (/uL)	CSF protein (mg/dL)	CSF glucose (mg/dL)	IV antiviral used	IV antiviral dosage	IV antiviral days	Oral antiviral used	Oral antiviral dosage	Oral antiviral days	Total antiviral days	Length of stay (days)	Patient outcome
Alataby 2020^9^	40	Male	None	T10	28	309	139	n/a	Acyclovir	1000 mg q8h	4	Valacyclovir	1000 mg q8h	14	18	7	Resolved
Alexakis 2024 (Case 4)^10^	47	Male	Multiple sclerosis; hypertension	n/a	n/a	n/a	n/a	n/a	Acyclovir	n/a	2	Acyclovir	n/a	12	14	n/a	Resolved
Alexakis 2024 (Case 7)^10^	44	Female	None	n/a	n/a	n/a	n/a	n/a	Acyclovir	n/a	9	Acyclovir	n/a	5	14	n/a	Resolved
Alexakis 2024 (Case 12)^10^	69	Female	Connective tissue disorder; hypertension; dyslipidemia	n/a	n/a	n/a	n/a	n/a	Acyclovir	n/a	14	Acyclovir	n/a	7	21	n/a	Resolved
Bhandari 2021 (Case 3)^11^	60	Female	Diabetes; schizoaffective disorder	n/a	n/a	106	71	90	Acyclovir	10 mg/kg q8h	n/a	Valacyclovir	n/a	n/a	n/a	n/a	Resolved
Chen 2020 (Case 2)^12^	22	Male	Childhood varicella	T10	17	200	57	70	Acyclovir	10 mg/kg q8h	10	None	n/a	0	10	10	Resolved
Chirumamilla 2023^13^	26	Male	Varicella; ADHD	S3-S4	n/a	174	136	48	Acyclovir	n/a	10	Valacyclovir	1000 mg q8h	4	14	10	Resolved
Cooper 2013^14^	47	Female	None	T4-T6	n/a	648	396	41	None	n/a	0	Acyclovir	800 mg q4h	13	13	3	Resolved
Faluk 2020^15^	45	Male	Hyperlipidemia	T10	n/a	741	296	41	Acyclovir	n/a	14	None	n/a	0	14	n/a	Resolved
Goyal 2013^16^	27	Male	None	C6-T1	n/a	50	n/a	n/a	Acyclovir	n/a	4	Acyclovir	n/a	14	18	4	Resolved
Imani 2020^17^	32	Male	Childhood varicella	L2	n/a	750	264	56	Acyclovir	10 mg/kg q8h	14	None	n/a	0	14	n/a	Resolved
Kangath 2012^18^	30	Female	MVP; anxiety/depression; tinea corporis; pneumonia	n/a	n/a	677	66	35	Acyclovir	n/a	7	Valacyclovir	n/a	14	21	7	Resolved
Khalil 2021^19^	24	Male	Varicella	n/a	n/a	717	302	41	Acyclovir	n/a	3	None	n/a	0	3	4	Resolved
Khaliq 2017^20^	28	Male	Childhood varicella	T4-T6	55	429	164	44	Acyclovir	n/a	n/a	Famciclovir	500 mg q8h	5	n/a	n/a	Resolved
Kushawaha 2009^21^	46	Female	Nephrolithiasis	T8-T9	n/a	127	125	49	None	n/a	n/a	Acyclovir	n/a	n/a	n/a	n/a	Resolved
Sohal 2020^22^	40	Male	Hypertension; migraine	n/a	n/a	121	106	51	Acyclovir	750 mg q8h	21	Acyclovir	1000 mg q8h	n/a	n/a	n/a	Resolved
Case Report	44	Male	Varicella; anxiety; allergic rhinitis	C4–C5	n/a	298	179	67	Acyclovir	10 mg/kg q8h	4	Valacyclovir	1000 mg q8h	10	14	6	Resolved

## Discussion

This pooled analysis of immunocompetent adults with PCR confirmed VZV meningitis and a spinal dermatomal rash highlights a clinically important, but underrecognized, subset of VZV neuroinfection. Across 17 patients the presentation was dominated by febrile headache with a concurrent dermatomal vesicular eruption. CSF analysis demonstrated a lymphocytic meningitis pattern with mean WBC 382/µL, protein 177 mg/dL, and glucose 52.7 mg. Intravenous acyclovir was administered in most cases with oral acyclovir or valacyclovir step-down, and full recovery occurred in all reported patients. We add purely cervical involved case within this pooled dataset, underscoring that cervical segments can be affected and that diagnosis may be delayed when the rash is initially subtle or misattributed.

### Prior VZV literature and rationale for a spinal-only analysis

Much of the modern VZV meningitis literature concentrates on cranial presentations, specifically trigeminal V1 (zoster ophthalmicus), where the clinical stakes, complications, and management differ from spinal involvement (Gilden et al. 2013; Kennedy 2023; Kennedy and Gershon 2018). In cranial, and particularly first-division (V1), disease the principal hazards are sight-threatening ocular complications (keratitis, uveitis, and acute retinal necrosis), cranial neuropathies, and a recognized association with VZV cerebral vasculopathy and delayed ischemic stroke; spinal-dermatomal disease is instead dominated by segmental complications such as motor radiculopathy or segmental weakness and, in sacral cases, neurogenic bladder, while vision loss and large-vessel stroke are uncommon. By excluding V1–V3 and restricting to immunocompetent adults with spinal dermatomes, the current review reduces heterogeneity and delineates a subset that appears to have a consistent CSF inflammatory profile, a high PCR diagnostic yield, a rapid response to antiviral therapy, and excellent short-term outcomes. This contrasts with broader VZV neuroinvasive cohorts where encephalitis, vasculopathy, or immunocompromise often drive more variable trajectories and residual deficits(Le Bot et al. 2021; Lee et al. 2021). Clinically, this narrower lens may help clinicians recognize that a spinal dermatomal eruption plus acute meningitis pattern strongly indicates a potential diagnosis of varicella zoster, even in young immunocompetent adults.

### Pathophysiology and anatomic considerations

VZV establishes latency in dorsal root ganglia(Nagel and Gilden 2014). Reactivation within a spinal ganglion can propagate along the sensory root to the skin, causing a rash, and also toward the thecal sac, leading to meningeal irritation(Gilden et al. 2015). The concurrence of dermatomal rash and meningitic symptoms likely reflects this radiculomeningeal spread, supported by the CSF inflammatory changes and the anatomic congruence of pain and headache with an ipsilateral segmental eruption(Reichelt et al. 2008). Cervical involvement, as in our case, may be under identified because clinicians more readily connect cranial dermatomes with CNS complications, whereas spinal rashes can be overlooked or attributed to contact dermatitis, insect bites, or shingles without CNS disease. The predominance of thoracic dermatomes and relative paucity of cervical cases may reflect true anatomic variability in ganglionic reactivation, reporting bias, or differences in clinical recognition(Kennedy 2023; Nemoto et al. 2024).

### Diagnostic implications

This pooled analysis brings several important diagnostic points to light. First, clinicians should not anchor diagnoses on rash alone. In several cases, meningitis was initially missed or misattributed when the rash was mild or evolving. A dermatomal eruption in the setting of febrile headache should prompt early lumbar puncture even when neuroimaging is normal(Liu et al. 2025; Miller et al. 2024). Second, CSF PCR is a necessity to confirm infection(Miller et al. 2024). The characteristic profile of pleocytosis, elevated protein, and preserved glucose overlaps with other viral meningitides, but VZV PCR positivity in CSF provides decisive confirmation(Chen et al. 2020). Lastly, it is important to remember that imaging is often unrevealing. Head CT/MRI were commonly normal or nonspecific. Absence of radiographic abnormalities should not delay empiric antiviral therapy when clinical suspicion is high. In summary, evidence for this systematic review justifies having an LP-first mindset in immunocompetent adults with compatible rash and meningitic symptoms. Deferring the tap risks delay in therapy (Nagel and Gilden 2014).

### Therapeutic considerations

Across the cohort, most patients received IV acyclovir and step-down to oral acyclovir or valacyclovir, with one report of use of famciclovir. While dosing and duration varied, the main shared features between cases were that antiviral therapy led to uniformly favorable outcomes. Early starting of therapy in relation to onset of symptoms is recommended to avoid complications and promote quicker recovery. Steroid use was rarely described and not necessary for recovery in this subset, thus adjunctive steroids therefore cannot be recommended as standard of care based on these data. We recommend starting IV acyclovir when suspicion is high, rather than waiting for PCR results. Transition to oral therapy is indicated once clinical improvement is noted and patients are able to tolerate it. Notably, emerging evidence suggests that outcomes in VZV meningitis may be favorable irrespective of the intensity of antiviral therapy: in a recent series of 123 PCR-confirmed cases, patients treated with oral valacyclovir alone or with no antiviral at all had outcomes equivalent to those receiving intravenous acyclovir (Dulin et al. 2024). Consistent with this, two patients in our pooled cohort received no intravenous antiviral yet recovered fully. Accordingly, while we favor prompt initiation of antiviral therapy, the choice between intravenous acyclovir and oral valacyclovir may reasonably be individualized in immunocompetent adults without encephalitic features.

### Outcomes and prognosis

A striking and clinically reassuring signal in this spinal-only, immunocompetent group is the 100% survival and full neurologic recovery reported. This suggests that absent encephalitic features, vasculopathy, or immunocompromise, in VZV meningitis with spinal dermatomal rash carries an excellent short-term prognosis when antivirals are initiated promptly. Length of stay, which was approximately 6 days, was modest, and readmissions or late complications were not described. The dataset is too small to quantify relapse risk, but none were reported in the included cases.

From a prevention standpoint, the recombinant zoster vaccine is highly effective against herpes zoster and its complications and is currently recommended by the Advisory Committee on Immunization Practices as two doses for immunocompetent adults aged 50 years and older and for immunosuppressed adults aged 19 years and older. Our patient, aged 44 and immunocompetent, fell below the recommended age and was unvaccinated. Cases of central nervous system zoster in adults younger than 50 highlight a small but real gap in the currently recommended vaccination window; however, given the low absolute incidence in this age group and the cost-effectiveness data underpinning the age-50 threshold, such cases do not by themselves justify broadening the recommendation. They nonetheless reinforce the importance of timely vaccination as adults reach eligibility.

### Cervical cases

Cervical dermatomes are less commonly emphasized in shingles teaching, yet they pose unique diagnostic pitfalls. Cervical involved cases, while less common than thoracic, can occur and may present with neck/retro-auricular rashes that are misattributed to bacterial cellulitis or tick-borne disease, which may lead to a delay starting appropriate antiviral therapy(Khalil and Hirashima 2021; Liu et al. 2025). Neck/shoulder pain and paresthesias can masquerade as musculoskeletal strain or cervical radiculopathy, leading to findings of scattered vesicles on the posterior neck or shoulder to possibly be dismissed. Our case demonstrates that cervical zoster can accompany meningitis without overt neurologic deficits or imaging abnormalities, and that careful skin examination, including hidden posterior dermatomes, can accelerate diagnosis.

### Practical takeaways and a suggested care pathway

Based on these data and our case experience, a streamlined approach is reasonable:

1. Recognize: acute febrile headache plus a dermatomal vesicular rash in a spinal distribution.
2. Act: obtain LP with CSF PCR for VZV; do not wait for imaging if there are no focal deficits or red flags.
3. Treat: initiate IV acyclovir (or oral valacyclovir in uncomplicated, non-encephalitic cases) while awaiting PCR results; avoid routine steroids in uncomplicated meningitis and use of unnecessary empiric antibiotics or antivirals.
4. Follow-up: step down to oral acyclovir or valacyclovir upon clinical improvement; see patient outpatient upon completion of antiviral course.
5. Document: record dermatome, timing of rash, symptom onset, CSF measures, antiviral dosing and duration, and outcome at discharge and follow-up.

### Strengths and limitations

Strengths include a focused inclusion strategy, patient level abstraction enabling pooled summaries, and inclusion of a well-documented contemporary case that extends dermatome representation. Limitations include small numbers of cases, incomplete data reporting (including the absence of a specified dermatome level in seven of 17 cases, which limits conclusions about the precise anatomic distribution), and potential publication bias toward favorable outcomes. Long-term neurocognitive outcomes could not be systematically assessed. Finally, by design we excluded cranial dermatomes so these conclusions should not be extrapolated to zoster ophthalmicus or to suspected VZV vasculopathy.

## Conclusion

VZV meningitis with spinal dermatomal rash occurs in immunocompetent adults and can involve cervical segments such as C4–C5. Recognition of the dermatome, prompt LP with PCR testing, and timely antiviral therapy are central to favorable outcomes. Our newly reported C4–C5 case expands the anatomic spectrum of cervical presentations and underscores how dermatomal localization can accelerate diagnosis and treatment.

## Data Availability

No datasets were generated or analysed during the current study.
